# Notes and News

**Published:** 1902-11-29

**Authors:** 


### NOTES AND NEWS.

His Majesty the King has sent a gift of pheasants to Queen Charlotte's Hospital, Marylebone Road, of which Queen Alexandra is Patron, and also to the Cancer Hospital, Brompton.

The first English lady to graduate in medicine at Leipzig is Miss E. Bloome, who has recently taken her degree as an M.D.

We are glad to learn that one of the insanitary station hospitals at Aldershot is to be closed. Provision for 300 patients has been provided for years in these wooden huts, which are most unsuitable for the purpose.

A law has been passed in Italy requiring employers of labour to supply their employees with quinine free when suffering from malaria, and the maximum price at which quinine can be sold to the public has been fixed.

Now is the time when the anti-vaccination ratepayer should pause and think. The Metropolitan Asylums Board has published its accounts, so to speak, for the expenditure on the recent outbreak of small-pox. In round figures this outbreak cost half a million of money. As outbreaks go this was a very small one. Will not the anti-vaccinists, when they have to pay the bill, consider whether the experiment, at any rate of prevention rather than cure, might well be tried on the grounds of economy, if for no other reason?

During the last two years the majority of cases of anthrax amongst wool workers have occurred amongst those working in Persian wools. The most recent example occurred in a man employed in a spinning mill, who had been working in Persian wool. The precautions taken met the requirements of the authorities. It would be interesting to know to what extent, if at all, the Persian wools are used by the manufacturers of "sanitary clothing." We have never regarded the popular use of half-stoved wools for underclothing without grave doubts.

A crematorium has been opened at Golder's Green by Sir Henry Thompson. It has been erected after the designs of Messrs. Ernest George and Yeates by the London Crematorium Company.

Dr. A. Kuliapko has been continuing his experiments in reanimating the heart. He has been lecturing at Moscow, and illustrating his lectures with successful experiments. Dr. Kuliapko's claim to this human power to re-establish the functions of the heart in a lifeless body should be studied and carefully watched.

The fatalities ascribed to broken hearts are usually associated with wounds to the affections, but the less poetic explanation is that they are mostly associated with the occupation of laundress. However, a beggar recently died in Pentonville Prison of rupture of the left auricle, thus sharing some of the romantic interest attaching to such deaths.

It would be wise if the proprietors of hotels and boarding houses in health resorts would consider more carefully the question of sanitary house decoration. It is not very comforting to find tapestry hanging on the walls in places which are specially recommended for diseases of the chest. The tuberculous germs would lurk in cosy corners of corridors and lounges. Paint, after all, can be quite as decorative and cheaper in the end, besides protecting the health of the public.

The battle of the northern universities still continues, and no solution to the difficulty has yet been arrived at. Whilst some of the graduates of the Victoria University are in favour of disbanding it, others object to the proposal to retain it as a local university for Manchester. The proposal to retain the Victoria as a medical university and allow Manchester, Liverpool, and Leeds each to have its own university for other subjects is also not favourably received.

Fortunately, the fire that broke out in the ironing-room of the laundry at the Royal Free Hospital, the news of which figured largely on certain evening newspaper placards on Saturday, was not extensive. The night porter, who had noticed nothing wrong when he made his round at 11 o'clock on Friday night, discovered the fire at a few minutes before midnight. He at once gave the alarm, and the staff got it under before the arrival of the local fire brigade. The laundry is separated by some 40 feet from the nearest ward, but as a purely precautionary measure about a dozen patients were removed. That there was no immediate cause for alarm is proved by the fact that while the inside of the ironing-room was entirely gutted and the glass roof destroyed, the washing room, separated by a brick wall, was untouched. The fire is supposed to have originated with the ironing-stove, close to the brick flue of which was a wooden cupboard. None of the patients suffered any injury, although, of course, there was some excitement for a short time before the fire was extinguished. The nurses and porters at the Royal Free Hospital are regularly drilled by an experienced fireman.

